# Tongue cancer microbial biomarkers: landscape of saliva, cancer tissue, and para-carcinoma tissue

**DOI:** 10.3389/fmed.2026.1817840

**Published:** 2026-06-04

**Authors:** Shuai Chen, Lei Wang, Bowen Yang, Xiaobo Dai, Chunjie Li, Bing Yan

**Affiliations:** 1 State Key Laboratory of Oral Diseases, Department of Head and Neck Oncology Surgery, National Center for Stomatology, National Clinical Research Center for Oral Diseases, West China Hospital of Stomatology, Sichuan University, Chengdu, China; 2 Department of Stomatology, The Second People's Hospital of Tibet Autonomous Region, Lhasa, China

**Keywords:** 16S rRNA sequencing, bacterial exosomes, non-invasive screening, tongue squamous cell carcinoma, tumor biomarkers

## Abstract

**Background:**

Oral cancer poses a significant global health challenge due to delayed intervention and a lack of effective early screening methods. About two-thirds of patients are diagnosed with advanced-stage at initial consultation. Tumor-associated bacteria are implicated in tissue carcinogenesis and have potential as diagnostic biomarkers.

**Methods:**

In this pilot study, 24 patients with tongue squamous cell carcinoma were enrolled. Saliva samples were collected from all participants before treatment. Cancer and para-carcinoma tissues were surgically obtained from 20 patients. Bacterial exosomes were extracted, and 16S rRNA sequencing was performed to characterize the microbial composition of saliva, cancer, and para-carcinoma tissue.

**Results:**

The microbial composition of cancer tissues, para-carcinoma tissues, and saliva from cancer patients showed significant differences compared to healthy individuals. Specifically, *acinetobacter calcoaceticus, dietzia natronolimnaea, burkholderia cepacia, and acinetobacter baumannii* were highly prevalent in cancer tissues but nearly absent in healthy individuals’ tissue. These microorganisms show promise as biomarkers for oral cancer. Additionally, *weissella cibaria, sphingomonas azotifigens*, and *aeromonas sobria* were more abundant in the saliva of TSCC patients compared to healthy individuals, highlighting their potential as non-invasive screening tools.

**Conclusion:**

This exploratory study identified microbial strains that are enriched in oral cancer tissues and saliva, highlighting their potential as non-invasive biomarker candidates for early detection. However, given the reliance on external healthy controls and limited sample size, these findings should be considered preliminary and require validation in future prospective studies with rigorously matched controls.

## Graphical abstract

**Figure:**
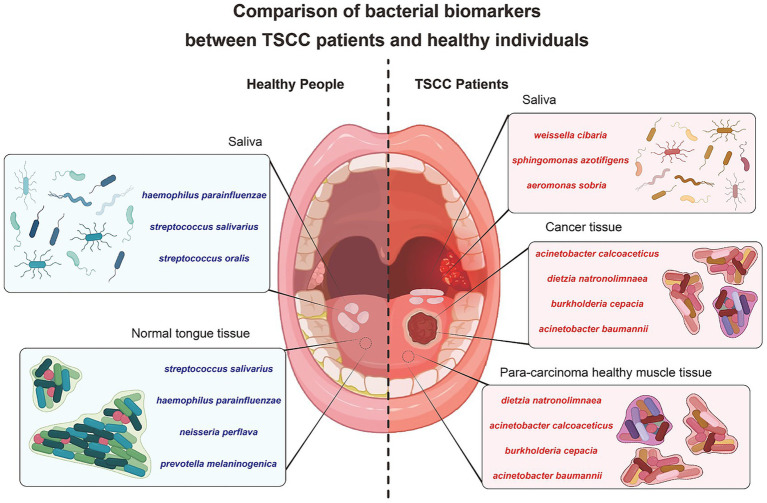

## 1 Introduction

Oral cancer poses a significant global public health challenge. In the United States, an estimated 58,450 new cases are expected in 2024 (2.9% of all new cancer cases) and resulting in approximately 12,230 deaths (2.0% of all cancer-related deaths) (1). Despite treatment advancements, the five-year survival rate for oral cancer remains around 60%, primarily due to late diagnoses and the aggressive nature of the disease (2). Early screening is crucial, yet incomplete techniques often lead to delayed diagnoses and poor prognoses. Tumor progression, invasion, and metastasis are influenced by both malignant cells and the tumor microenvironment (TME), which includes the tumor-associated microbiota (3). Recent studies have shed light on the role of these microbiota in cancer development and progression (4, 5). Tumor-associated microbiota impact various aspects of tumor biology, including immune evasion (6), metastasis (7, 8), and resistance to chemo-immunotherapy (9–11). Such bacteria are found in various cancer types, especially solid tumors (12, 13). Imaging and molecular techniques have identified bacteria within malignant cells’ cytoplasm, tumor necrosis sites, or the tumor core, showing specific spatial distribution (14, 15). Determining the boundary between cancerous and healthy tissue is crucial yet challenging in oral cancer treatment. Malignant tumors exhibit invasive growth, lack an intact capsule, and have irregular margins, complicating differentiation from normal tissue. This complexity is exacerbated by the potential for distant metastasis (16). Accurate delineation of cancer boundaries is essential for effective surgical resection and precise radiotherapy. Tumor-associated microbiota have emerged as promising markers for distinguishing malignant from normal tissues (17). While the microbiota in normal oral tissues and saliva is well-studied, there is limited research comparing bacterial communities in cancerous tissues with adjacent healthy tissues. Besides, non-invasive cancer diagnosis is vital for effective treatment and improved survival rates. Due to delayed early detection, about two-thirds of oral cancer patients are diagnosed with locally advanced disease at their first visit (18). Thus, there is a need for a non-invasive, convenient, and cost-effective method for early mass screening. Saliva, with its unique biomarkers, offers a promising tool for non-invasive cancer detection (19). Microbiological analysis of saliva is cost-effective and suitable for large-scale screening, reducing the risk of cancer metastasis (20). Saliva’s mobility results in different microbiota from the stable environment within oral tissues, and it contains abundant bacterial exosomes. Sequencing the microbial environment from saliva can develop a non-invasive early screening program to identify high-risk groups for oral cancer. Measuring bacterial exosomes in tissues and saliva aids in early detection, monitoring tumor progression, and assessing treatment responses (21).

In this study, we conducted microbiota profiling in TSCC cancerous tissue (CT), para-carcinoma healthy muscle tissue (PT), and cancer patient’s saliva (CS). We compared these profiles with those from normal person’s oral tissues (NT) and saliva (NS) to identify potential microbial biomarkers. We also conducted a comparative analysis of the microbiota in CT, PT, and NT to assess the potential involvement of microbial flora in cancer development. Our goal is to identify candidate markers that can distinguish between cancerous tissue, high-risk tissue, and healthy tissue. Additionally, we aim to facilitate non-invasive early screening and cancer risk assessment through the comparative analysis of microbial communities in the saliva of cancer patients and healthy individuals.

## 2 Materials and methods

### 2.1 Sample collection of subjects

From January to May 2024, we included 24 patients diagnosed with malignant tongue tumors at the Department of Head and Neck Oncology, West China Stomatological Hospital, Sichuan University, China. Inclusion criteria were: (i) age > 18 years; (ii) no other diseases (other oral diseases, diabetes, hypertension); (iii) TSCC diagnosis confirmed by CT and pathology; (iv) no antibiotic use in the past month; (v) no prior surgery, chemotherapy, physiotherapy, targeted therapy, or immunotherapy; (vi) no family history of cancer; and (vii) eligibility for surgery per CSCO 2024 TSCC Guidelines. The first 20 patients underwent surgery, during which both TSCC tissues and adjacent healthy muscle tissues (0.5 cm × 0.5 cm) were resected. The remaining 4 patients, who declined surgery, only provided saliva samples. All tissue specimens were collected immediately after tumor resection under sterile conditions in the operating room. Sampling was performed with strict avoidance of necrotic areas and electrocautery burn zones to preserve tissue viability and the *in situ* authenticity of the microbiota. Each specimen was immediately placed into a sterile cryovial, rapidly transported on ice to the laboratory, snap-frozen in liquid nitrogen, and stored. Exosome extraction was initiated after thawing of all collected specimens to minimize post-collection changes in microbial composition. All sampling instruments were sterile, and the operator wore sterile gloves throughout the procedure to ensure contamination-free handling. Saliva samples were collected preoperatively in the early morning after overnight fasting. Following a mouth rinse with sterile saline, unstimulated whole saliva was allowed to flow naturally into a sterile collection tube, with a volume of approximately 2–5 mL. The saliva samples were immediately placed on ice and transported to the laboratory for subsequent processing. The study adhered to the ethical guidelines of the 1975 Declaration of Helsinki, with approval from the Institutional Human Research Committee. Patient data were encoded for confidentiality and managed according to GDPR. Informed consent was obtained from all participants.

### 2.2 Bacterial exosome extraction and characterisation

Samples were thawed at 37 °C and centrifuged at 2000 × g for 30 min at 4 °C. The supernatant was transferred to a new tube and centrifuged at 10000 × g for 45 min at 4 °C. After filtering the supernatant through a 0.45 μm membrane, the filtrate was centrifuged at 100000 × g for 70 min at 4 °C. The resulting pellet was suspended in 10 mL of pre-cooled 1 × PBS. Aliquots of 20 μL were used for electron microscopy, 10 μL for particle size measurement, and 10 μL for sterility testing. The remaining sample was prepared for DNA sequencing. For sterility assurance, 5 mL of LB medium was inoculated with 10 μL of the exosome suspension and incubated at 37 °C for 24 h. Exosome samples were placed on a copper grid for 1 min, with excess liquid removed by filter paper. The grid was then treated with 10 μL of dioxycycloamyl acetate for 1 min, followed by drying at room temperature. Transmission electron microscopy (TEM) was conducted at 100 kV to examine the samples.

### 2.3 16S rRNA gene sequencing

Genomic DNA was extracted using CTAB or SDS and quantified by Nanodrop. Purity and integrity were confirmed by 1% agarose gel electrophoresis. DNA was diluted to 1 ng/μL with sterile water. Specific primers with barcodes were used to amplify the 16S/18S rRNA gene in 30 μL PCR reactions containing 15 μL of high-fidelity PCR master mix, 0.2 μM of each primer, and 10 ng of template DNA. Cycling conditions included an initial denaturation at 98 °C for 1 min, 30 cycles of 98 °C for 10 s, 50 °C for 30 s, and 72 °C for 30 s, followed by a final extension at 72 °C for 5 min. PCR products were mixed with 1 × SYBR green loading buffer and analyzed on a 2% agarose gel. Amplicons showing 400–450 bp bands were pooled and purified using the TIANgel purification kit. The purified amplicons were used to prepare Illumina DNA libraries with the TIANSeq Fast DNA library preparation kit. Library quality was assessed by Qubit 2.0 Fluorometer and Agilent Bioanalyzer 2,100. Sequencing was performed on an Illumina platform using a 2 × 250 bp paired-end protocol.

### 2.4 Data analysis

Microbiome bioinformatics analysis was conducted using QIIME 2 with DADA2 denoising. Sequence data were processed with the demux plugin and primers trimmed using cutadapt. Sequences were quality-filtered, denoised, merged, and chimeras removed via DADA2. Taxonomic annotations were assigned using the Greengenes_13_8 database for 16S rRNA, SILVA_138 for 18S rRNA, and Unite for the ITS region. Phylogenetic relationships were constructed with multiple sequence alignments in QIIME2, analyzing ASV relationships and dominant species differences. Alpha diversity was assessed using observed species, Nest, Shannon, Simpson, ACE, and Good’s coverage indices with QIIME2 and R software. Beta diversity was calculated using weighted and unweighted UniFrac metrics and visualized through principal coordinate analysis (PCoA) in QIIME2 and R (ade and ggplot2 packages). Statistical analysis to distinguish classifications or functional annotation differences employed Metastats, STAMP, and LEfSe (LDA score>4). Bray–Curtis dissimilarity-based ANOVA was used to assess microbial community differences between groups. For comparative analyses involving healthy individuals, we utilized publicly available microbial profiles from the expanded Human Oral Microbiome Database (eHOMD).^1^ The eHOMD is a well-curated reference database that provides comprehensive taxonomic information on the oral microbial communities of healthy individuals. We retrieved healthy reference data for normal oral tissue and normal saliva from the database, selecting entries that matched our study in terms of sample type and general demographic characteristics where available. These reference data were used to compare the microbial composition and relative abundance of taxa between TSCC patient samples (CT, PT, CS) and healthy controls (NT, NS).

## 3 Results

### 3.1 Clinical baseline characteristics of participants

**Figure 1:**
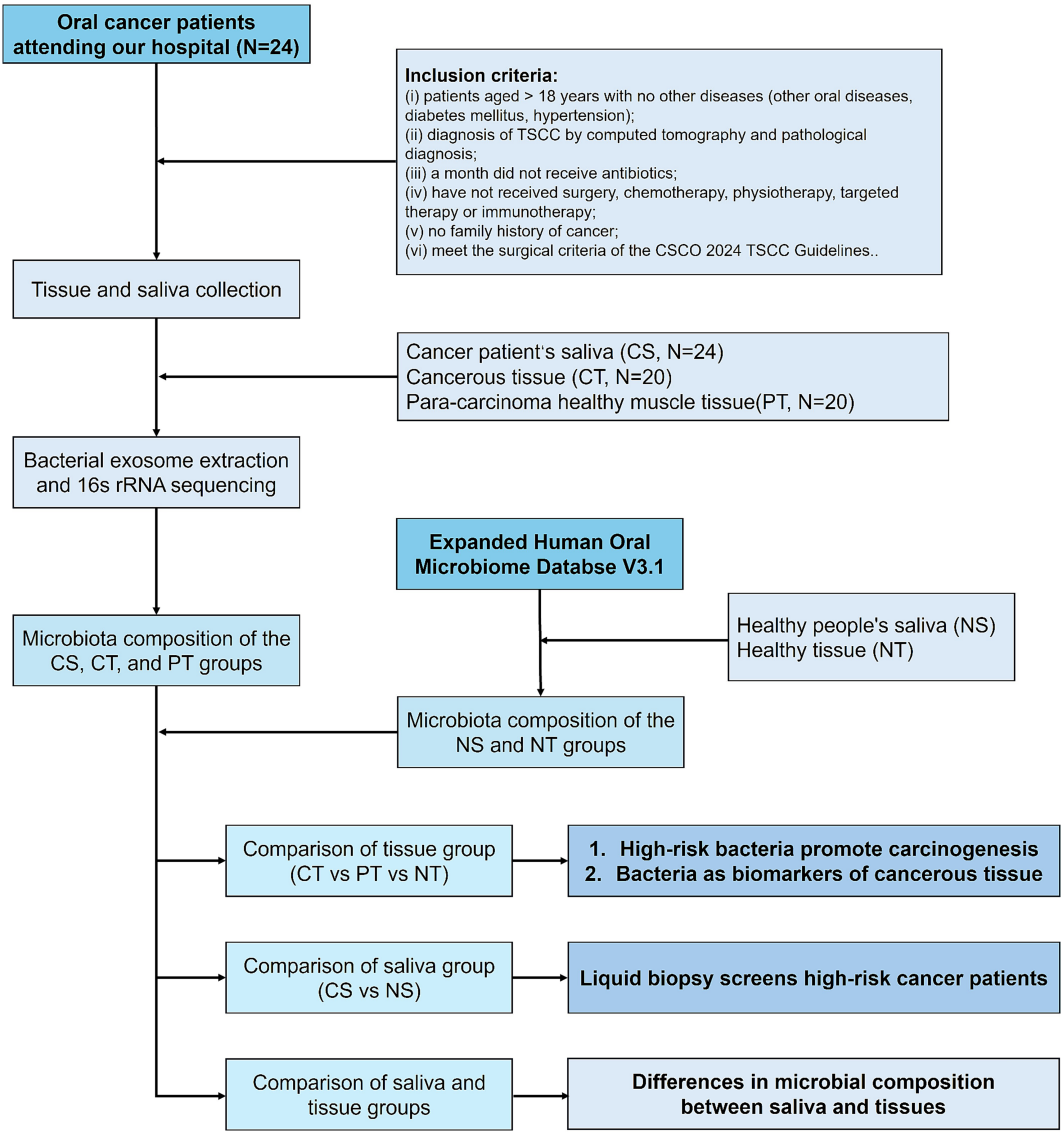
Research flow chart.

The study included 24 subjects, averaging 60 years old (range 32–82), with 11 women (45.83%) and 13 men (54.17%). Distribution by stage was: 4 in stage I (16.67%), 9 in stage II (37.5%), 5 in stage III (20.83%), and 6 in stage IV (25%; Supplementary Table 1) None had received antibiotics in the past month. All patients were newly diagnosed with TSCC and had not undergone any prior treatments (Figure 1).

### 3.2 Characterisation of bacterial exosomes extracted from TSCC patients

**Figure 2:**
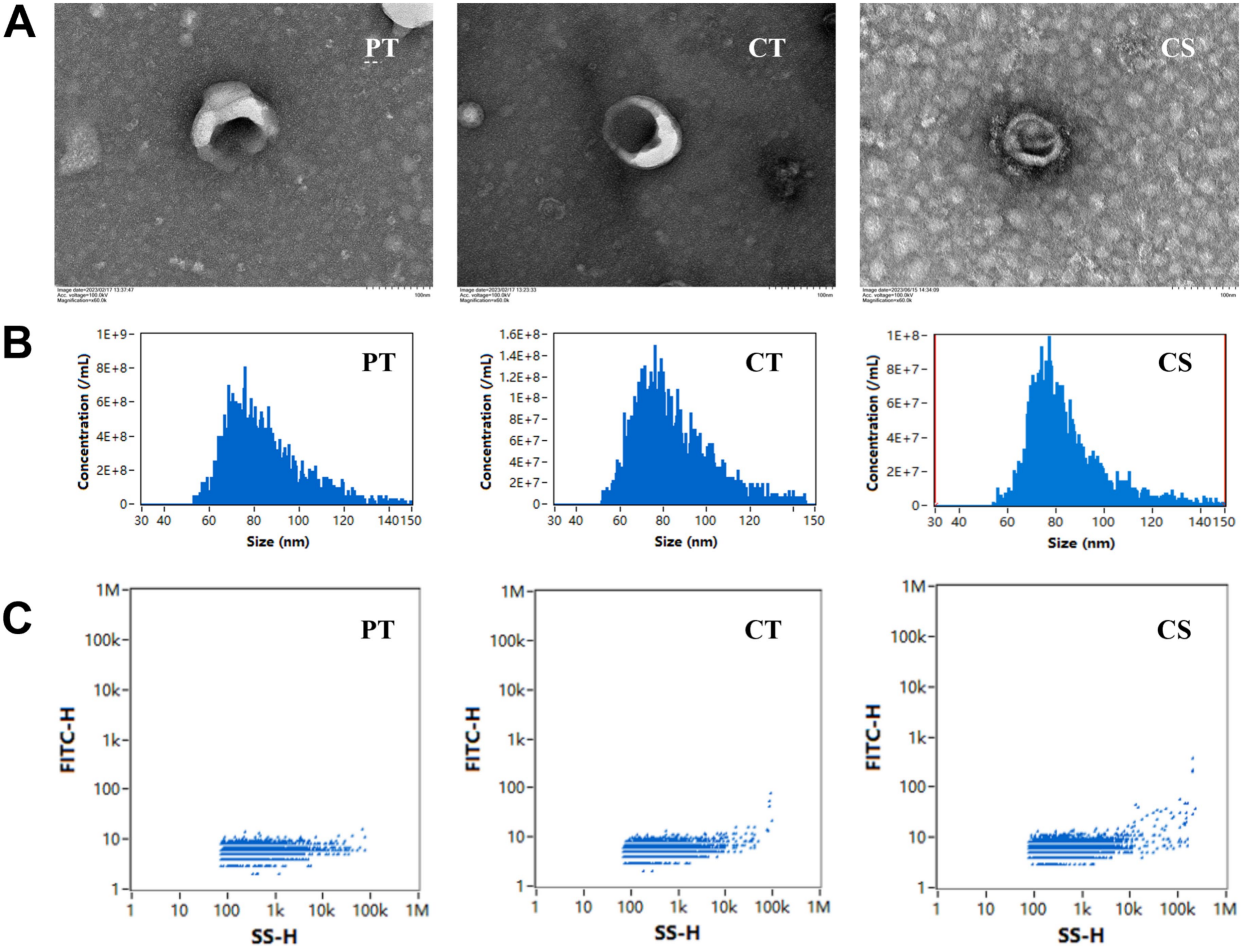
Bacterial exosomes isolated from TSCC patients. **(A)** TEM images confirmed the characteristic vesicle shape of exosomes from TSCC patients. Scale bar = 100 nm. **(B)** Nano-flow cytometry size distribution histogram showed most exosomes were 70–90 nm. **(C)** Scatter plots indicated consistent exosome concentrations across various sample types.

Bacterial exosomes were isolated from CT, PT, and CS groups. TEM confirmed goblet-shaped vesicles in all samples. Nanoflow cytometry showed no significant differences in size, concentration, or purity among the groups. These consistent exosome profiles suggest potential for non-invasive diagnostics, as uniform bacterial exosomes were successfully isolated from cancer tissues, adjacent healthy tissues, and saliva (Figure 2).

### 3.3 Comparative analysis of TSCC microbial diversity

**Figure 3:**
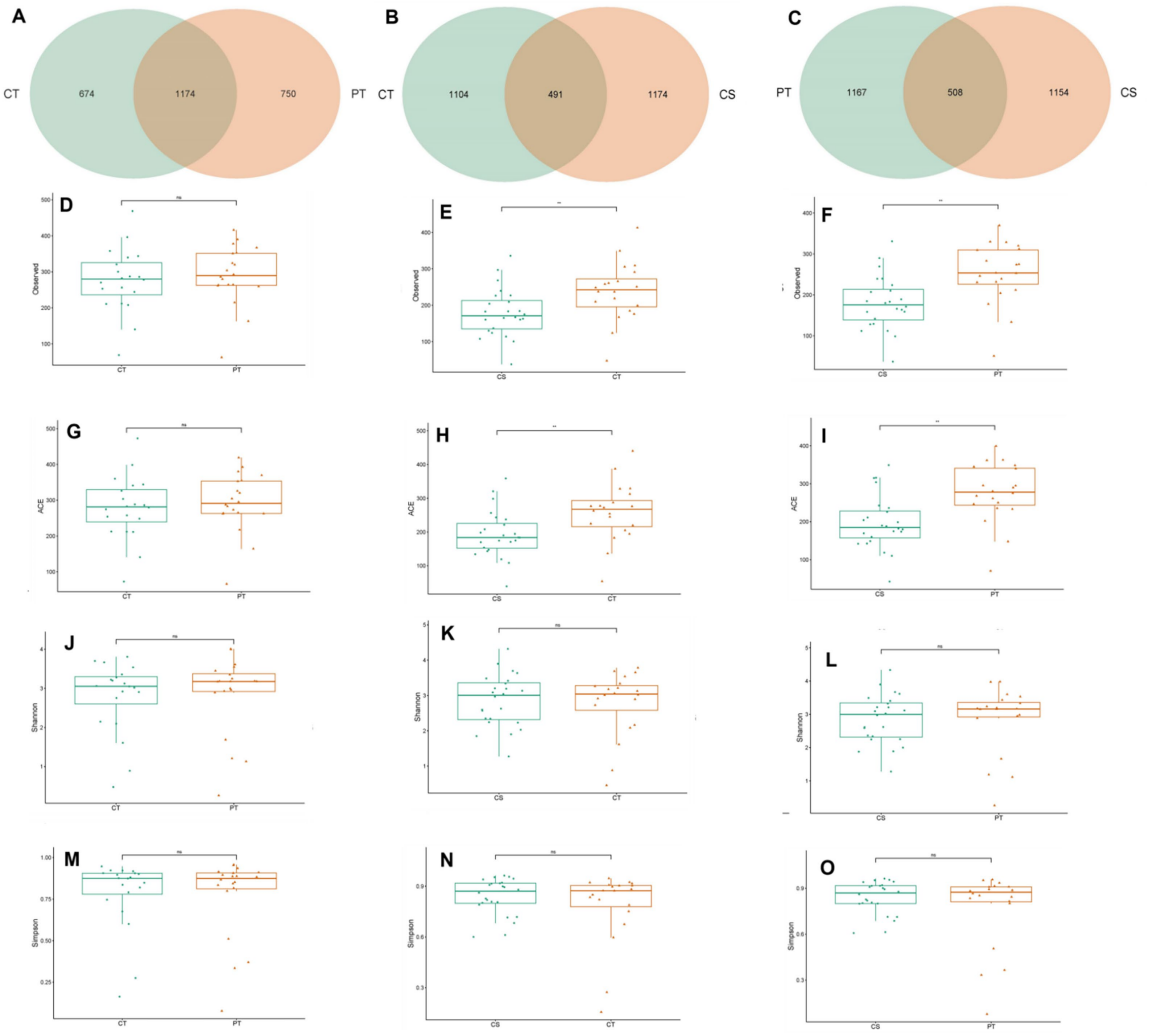
Comparative analysis of TSCC microbiota alpha diversity. **(A–C)** Venn diagrams show OTU overlap and distinctiveness among the three groups. **(D–F)** Observed indices indicated no significant difference between CT and PT, but CS indices were significantly lower than CT and PT (*p* < 0.01). GI ACE index comparisons showed no significant difference between CT and PT, but CS ACE index was significantly lower than CT and PT (*p* < 0.01). **(J–O)** Shannon and Simpson’s index comparisons revealed no significant differences among the three groups.

**Figure 4:**
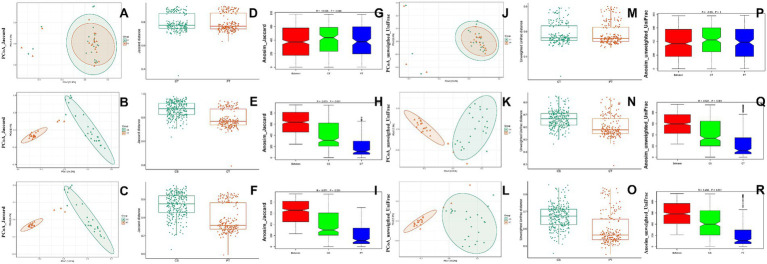
Beta diversity analysis of TSCC patients’ microbial communities. **(A–C)** Jaccard_PcoA assessed beta diversity in CT, PT, and CS groups. **(D,E)** Jaccard distances highlighted microbial community differences among CT, PT, and CS. **(G,H)** ANOSIM based on Jaccard distance showed no significant differences between CT and PT (*R* = −0.058, *p* = 0.098), but significant differences between CS and both CT (*R* = 0.673, *p* = 0.001) and PT (*R* = 0.671, *p* = 0.001). **(J–L)** Unweighted UniFrac PCoA evaluated beta diversity changes in CT, PT, and CS, incorporating phylogenetic distances. **(M–O)** Unweighted UniFrac distances compared CT, PT, and CS, reflecting phylogenetic community divergences. **(P–R)** ANOSIM using unweighted UniFrac distance found no significant difference between CT and PT (*R* = −0.06, *p* = 1), but higher distances for CS compared to CT (*R* = 0.535, *p* = 0.001) and PT (*R* = 0.496, *p* = 0.001).

Venn diagrams illustrated the shared and unique operational taxonomic units (OTUs) among CT, PT, and CS groups. PT and CT groups had 1924 and 1848 OTUs respectively, with 1,174 overlapping. CS and CT groups had 1,665 and 1,595 OTUs respectively, with 491 overlapping. CS and PT groups had 1,662 and 1,675 OTUs respectively, with 508 overlapping, indicating high similarity between PT and CT but distinct differences with CS. Alpha and beta diversity analysis showed distinct microbial profiles among the groups. No significant differences in the Shannon and Simpson indices were found among CT, PT, and CS groups. However, the Observed ACE index for CS was significantly lower than CT (*p* < 0.05) and PT (*p* < 0.01; Figure 3) ANOSIM revealed greater microbial community similarity between CT and PT groups than with the CS group, supported by Jaccard and Unweighted UniFrac analyses, which showed no significant differences between CT and PT, but significant dissimilarities between CS and CT (Jaccard_Anosim: R = 0.673, *p* = 0.001; UniFrac_Anosim: R = 0.535, *p* = 0.001) and CS and PT (Jaccard_Anosim: R = 0.671, *p* = 0.001; UniFrac_Anosim: R = 0.496, *p* = 0.001; Figure 4).

### 3.4 Comparative analysis of microbial composition within the tissue group and within the saliva group

We compared the microbiota composition in CT, PT, NT, CS, and NS groups, focusing on the top 10 taxa at six taxonomic levels. These differences suggest potential biomarkers for malignancy susceptibility and early non-invasive screening.

**Figure 5:**
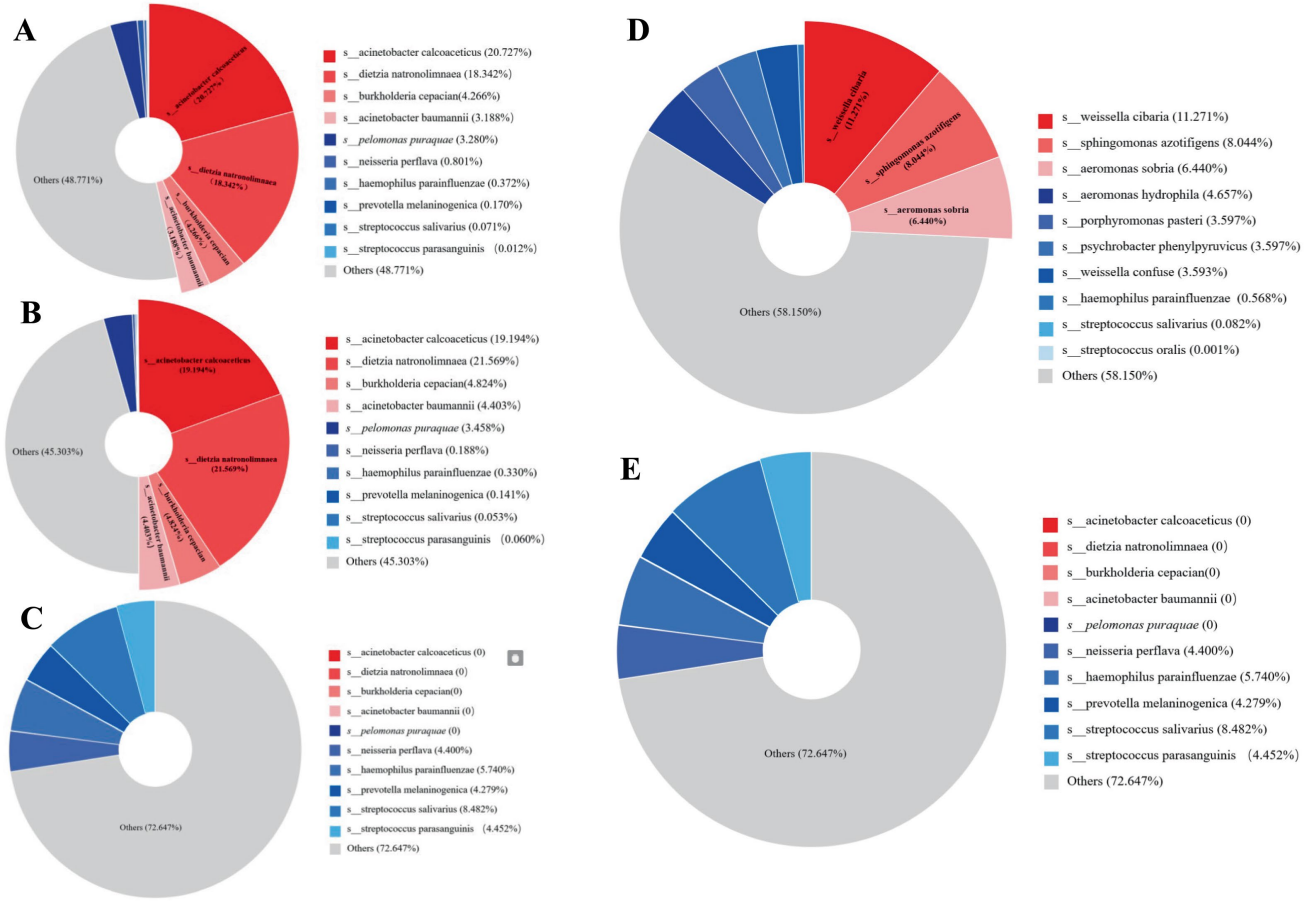
Comparative analysis of microbial composition in tissue (CT, PT, and NT) and salivary groups (CS and NS). Top 10 bacterial taxa by relative abundance at the genus level for tissue groups (CT, PT, NT) in panels **(A–C)**, and for salivary groups (CS, NS) in panels **(D,E)**.

In tissue samples, CT and PT groups exhibited high similarity in microbiota composition, whereas significant differences were observed with NT. Proteobacteria were significantly more abundant in CT and PT than in NT (CT: 66.203%; PT: 61.392%; NT: 12.405%), while Firmicutes were more prevalent in NT (CT: 6.056%; PT: 7.021%; NT: 35.680%), suggesting these phyla may serve as distinguishing indicators. At the class level, Gammaproteobacteria and Coriobacteriia were abundant in CT and PT but nearly absent in NT, which instead had higher levels of Bacilli. At the order level, CT and PT showed high levels of Pseudomonadales, Actinomycetales, and Burkholderiales, while NT was higher in Lactobacillales and Bacteroidales. At the family level, *Moraxellaceae*, *Dietziaceae*, and *Burkholderiaceae* were prominent in CT and PT, whereas *Streptococcaceae* and *Prevotellaceae* dominated in NT. At the genus level, CT and PT were rich in *Acinetobacter*, *Dietzia*, and *Burkholderia*, while NT had higher levels of *Streptococcus* and *Prevotella*. Species-specific observation revealed *dietzia natronolimnaea*, *acinetobacter calcoaceticus*, *burkholderia cepacia*, and *acinetobacter baumannii* were exclusive to CT and PT, while *streptococcus salivarius*, *haemophilus parainfluenzae*, and *streptococcus parasanguinis* were more prevalent in NT (Figure 5; Table 1).

**Table 1:** Comparative analysis of microbial composition in tissue and salivary groups.

Taxa	Bacterial names	Relative abundance (%)			Taxa	Bacterial names	Relative abundance (%)	
		CT	PT	NT			CS	NS
Phylum	p__Proteobacteria	66.203	61.392	12.405	Phylum	p__Proteobacteria	55.708	12.061
	p__Firmicutes	6.056	7.021	35.680		p__Firmicutes	34.754	34.647
	p__Actinobacteria	22.749	27.138	7.424		p__Bacteroidetes	4.166	15.903
	p__Bacteroidetes	4.376	4.061	12.421		p__Actinobacteria	4.331	4.258
	p__Fusobacteria	0.247	0.144	6.304		p__Fusobacteria	0.324	4.071
	p__TM7	0.048	0.050	0.654		p__ TM7	0.189	3.221
	p__SR1	0.118	0.036	0.026		p__Spirochaetes	0.408	0.190
	p__Spirochaetes	0.109	0.018	0.028		p__ SR1	0.001	0.082
	p__Verrucomicrobia	0.041	0.071	0.000		p__Verrucomicrobia	0.047	0.000
	p__Planctomycetes	0.003	0.022	0.000		p__Deferribacteres	0.034	0.000
Class	c__Gammaproteobacteria	41.454	37.189	4.498	Class	c__Bacilli	31.302	21.123
	c__Coriobacteriia	22.749	27.137	0.648		c__Gammaproteobacteria	29.068	4.617
	c__Bacilli	4.045	5.791	25.027		c__Alphaproteobacteria	22.880	0.000
	c__Betaproteobacteria	20.735	20.010	6.871		c__Bacteroidia	0.995	14.819
	c__Bacteroidia	1.975	1.113	11.921		c__Negativicutes	0.000	8.976
	c_Negativicutes	0.000	0.000	7.152		c__Betaproteobacteria	2.820	5.097
	c_Actinomycetia	0.000	0.000	6.776		c__Coriobacteriia	4.331	0.651
	c_Fusobacteriia	0.247	0.144	6.304		c__Clostridia	2.343	4.123
	c_Alphaproteobacteria	3.870	4.058	0.000		c__Fusobacteriia	0.324	4.071
	c_Clostridia	1.969	1.182	3.167		c__Actinomycetia	0.000	3.607
Order	o__Pseudomonadales	36.598	32.200	0.000	Order	o__Lactobacillales	22.093	20.270
	o__Actinomycetales	22.628	27.081	4.764		o__Bacteroidales	0.995	14.819
	o_Lactobacillales	3.484	3.930	24.258		o__Aeromonadales	14.815	0.000
	o__Burkholderiales	16.520	17.807	0.045		o__Sphingomonadales	12.110	0.000
	o_Bacteroidales	1.975	1.113	11.921		o__Rhizobiales	9.682	0.000
	o_Veillonellales	0.000	0.000	6.987		o__Bacillales	9.109	0.854
	o_Neisseriales	4.024	1.974	6.826		o__Pseudomonadales	8.881	0.007
	o_Fusobacteriales	0.247	0.144	6.304		o__Veillonellales	0.000	7.391
	o_Pasteurellales	1.268	1.329	4.497		o__Pasteurellales	0.665	4.604
	o_Eubacteriales	0.000	0.000	3.167		o__Neisseriales	0.232	4.307
Family	*f__Moraxellaceae*	28.383	27.343	0.000	Family	*f__Leuconostocaceae*	18.905	0.000
	*f__Streptococcaceae*	0.003	0.002	22.279		*f__Streptococcaceae*	0.550	18.752
	*f__Dietziaceae*	18.343	21.570	0.000		*f__Aeromonadaceae*	14.815	0.091
	*f__Burkholderiaceae*	10.750	11.874	0.045		*f__Sphingomonadaceae*	11.727	0.000
	*f__Prevotellaceae*	0.003	0.001	9.279		*f__Prevotellaceae*	0.189	10.482
	*f__Pseudomonadaceae*	8.215	4.857	0.000		*f__Veillonellaceae*	0.315	7.391
	*f__Veillonellaceae*	1.083	0.580	6.987		*f__Moraxellaceae*	7.390	0.006
	*f__Neisseriaceae*	4.024	1.974	6.826		*f__Bacillaceae*	6.794	0.000
	*f__Comamonadaceae*	5.097	5.196	0.000		*f__Pasteurellaceae*	0.665	4.604
	*f__Actinomycetaceae*	0.198	0.319	4.764		*f__Neisseriaceae*	0.232	4.307
Genus	*g__Acinetobacter*	27.232	26.275	0.000	Genus	*g__Streptococcus*	0.355	18.752
	*g__Streptococcus*	1.209	1.404	22.279		*g__Weissella*	18.510	0.000
	*g__Dietzia*	18.343	21.570	0.000		*g__Aeromonas*	14.787	0.000
	*g__Prevotella*	0.766	0.453	8.646		*g__Sphingomonas*	11.002	0.000
	*g__Pseudomonas*	8.160	4.834	0.000		*g__Prevotella*	0.185	9.424
	*g__Burkholderia*	6.627	7.407	0.000		*g__Veillonella*	0.045	6.906
	*g__Neisseria*	3.392	1.816	6.802		*g__Bacillus*	6.668	0.000
	*g__Veillonella*	0.629	0.446	6.728		*g__Psychrobacter*	6.009	0.000
	*g__Haemophilus*	0.497	0.471	4.466		*g__Haemophilus*	0.578	4.311
	*g__Ralstonia*	3.998	4.309	0.000		*g__Neisseria*	0.226	4.210
Species	*s__dietzia natronolimnaea*	18.342	21.569	0.000	Species	*s__weissella cibaria*	11.271	0.000
	*s__acinetobacter calcoaceticus*	20.727	19.194	0.000		*s__sphingomonas azotifigens*	8.044	0.000
	*s__streptococcus salivarius*	0.071	0.053	8.482		*s__aeromonas sobria*	6.440	0.000
	*s__haemophilus parainfluenzae*	0.372	0.330	5.740		*s__haemophilus parainfluenzae*	0.568	4.948
	*s__burkholderia cepacia*	4.266	4.824	0.000		*s__streptococcus salivarius*	0.082	4.919
	*s__streptococcus parasanguinis*	0.012	0.060	4.452		*s__aeromonas hydrophila*	4.657	0.000
	*s__acinetobacter baumannii*	3.188	4.403	0.000		*s__porphyromonas pasteri*	3.597	4.186
	*s__neisseria perflava*	0.801	0.188	4.400		*s__streptococcus oralis*	0.001	4.040
	*s__prevotella melaninogenica*	0.170	0.141	4.279		*s__psychrobacter phenylpyruvicus*	3.597	0.000
	*s__pelomonas puraquae*	3.280	3.458	0.000		*s__weissella confusa*	3.593	0.000

The table presents the top 10 bacterial taxa with the highest relative abundances across six taxonomic levels for the tissue groups (CT, PT, NT) and salivary groups (CS, NS). Data for CT, PT, and CS groups were sourced from TSCC patients at our hospital, while NT and NS data were derived from the expanded Human Oral Microbiome Database (eHOMD).

In saliva samples, significant differences in microbial composition were observed between CS and NS, indicating potential for early non-invasive screening. Proteobacteria were significantly more abundant in CS (CS: 55.708%; NS: 12.061%), whereas Bacteroidetes were more prevalent in NS (CS: 4.166%; NS: 15.903%). At the class level, Gammaproteobacteria and Alphaproteobacteria predominated in CS, while Bacilli were abundant in both groups. Bacteroidia and Negativicutes were primarily found in NS. At the order level, CS was dominated by Aeromonadales, Sphingomonadales, Rhizobiales, Bacillales, and Pseudomonadales, while NS had higher levels of Bacteroidales and Veillonellales. At the family level, *Leuconostocaceae*, *Aeromonadaceae*, and *Sphingomonadaceae* were exclusive to CS, whereas *Streptococcaceae*, *Prevotellaceae*, and *Veillonellaceae* were more common in NS. At the genus level, *Weissella*, *Aeromonas*, and *Sphingomonas* were unique to CS, while *Streptococcus*, *Prevotella*, and *Veillonella* dominated in NS. Species-specific findings revealed *weissella cibaria*, *sphingomonas azotifigens*, and *aeromonas sobria* were exclusive to CS, whereas *haemophilus parainfluenzae* and *streptococcus salivarius* were predominantly found in NS (Figure 5; Table 1).

### 3.5 Comparative analysis of microbial composition in CT, PT, and CS groups

**Figure 6:**
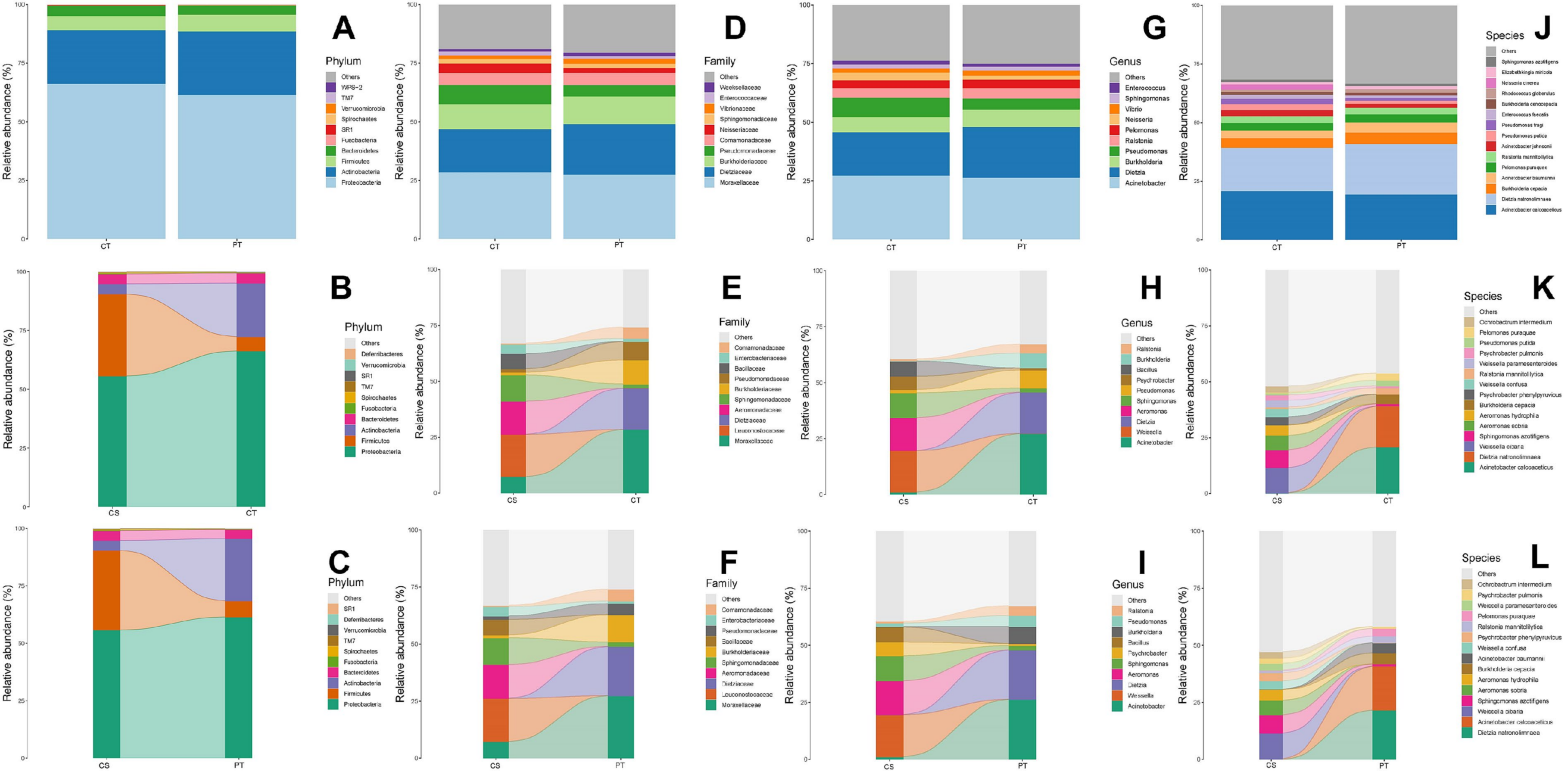
Relative abundance of the top 10 microbiomes at different taxonomic levels. Relative abundance values of the top 10 bacterial taxa in exosomes from three sample types (CT, PT, CS) are depicted across four taxonomic levels: phylum **(A–C)**, family **(D–F)**, genus **(G–I)**, and species **(J–L)**.

We analyzed the microbial composition of CT, PT, and CS from TSCC patients, focusing on the top 10 bacterial taxa by relative abundance at the phylum, family, genus, and species levels (Figure 6). At the phylum level, the predominant taxa were Proteobacteria (CT: 66.2%; PT: 61.4%; CS: 55.7%), Actinobacteria (CT: 22.7%; PT: 27.1%; CS: 4.3%), Firmicutes (CT: 6.1%; PT: 7.0%; CS: 34.8%), and Bacteroidetes (CT: 4.4%; PT: 4.1%; CS: 4.2%). Actinobacteria were significantly higher in CT and PT compared to CS, while Firmicutes were more prevalent in CS. The F/B ratios were higher in CS (CT: 1.38; PT: 1.73; CS: 8.34). At the family level, *Leuconostocaceae*, *Aeromonadaceae*, *Sphingomonadaceae*, and *Bacillaceae* were significantly more abundant in CS, whereas *Moraxellaceae*, *Dietziaceae*, *Burkholderiaceae*, *Pseudomonadaceae*, and *Comamonadaceae* were more abundant in CT and PT. Genus-level analysis showed *Weissella*, *Aeromonas*, *Sphingomonas*, *Psychrobacter*, and *Bacillus* were prevalent in CS, whereas *Acinetobacter*, *Dietzia*, *Pseudomonas*, and *Burkholderia* were higher in CT and PT. At the species level, *weissella cibaria*, *sphingomonas azotifigens*, *aeromonas sobria*, and *aeromonas hydrophila* were predominant in CS. Conversely, *dietzia natronolimnaea* and *acinetobacter calcoaceticus* were more prevalent in CT and PT (Table 1).

## 4 Discussion

Our study found that the microbiota composition of TSCC cancerous tissues closely resembled that of adjacent healthy muscle tissue, with minor differences, but significantly differed from normal dorsal tongue tissues. This similarity may indicate a potential predisposing mechanism for cancer risk. Predominant bacteria in cancerous and para-carcinoma tissues included Proteobacteria, Gammaproteobacteria, and specific species such as *acinetobacter calcoaceticus*, *burkholderia cepacia*, *acinetobacter baumannii,* and *dietzia natronolimnaea*, which were rare or absent in normal tissues. Notably, the pathogenicity and inflammatory implications of *acinetobacter calcoaceticus*, *burkholderia cepacia*, and *acinetobacter baumannii* in TSCC patients suggest their potential as biomarkers for oral cancer. These findings highlight the complex role of microbiota in cancer pathogenesis and warrant further investigation (22, 23) (Supplementary Figures 1, 2).

The microbial communities of cancer tissues and para-carcinoma tissues were highly similar, whereas saliva samples formed a distinct cluster separate from both tissue groups. This pattern reflects the inherent ecological niche differences between tissue-resident and salivary microbiota. Tissue-adherent communities are shaped by local selective pressures such as host immune surveillance, nutrient availability within the tumor microenvironment, and direct epithelial interaction, which may favor the colonization and persistence of specific taxa. In contrast, salivary microbiota represent a composite of microorganisms shed from various oral surfaces, subjected to the flushing effects of salivary flow and the dynamic conditions of the oral cavity. Given these fundamental differences, we focused our comparative strategy on within-subsite analyses—comparing cancer and para-carcinoma tissues against healthy tissues, and cancer patient saliva against healthy saliva—to minimize the confounding effect of niche divergence. We recognize that a more systematic comparison across subsites, including the identification of taxa with differential abundance patterns unique to each compartment, represents an important direction for future research. With larger sample sizes and dedicated study designs, it will be possible to disentangle the contributions of niche-specific selection from disease-associated alterations and to evaluate whether combined multi-subsite signatures offer improved diagnostic performance over single-compartment biomarkers.

Traditional studies have implicated *porphyromonas gingivalis*, *fusobacterium nucleatum*, *Prevotella* spp., *Streptococcus* spp. and *capnocytophaga gingivalis* in oral cancer development (24, 25). Our study identifies *acinetobacter calcoaceticus, dietzia natronolimnaea, burkholderia cepacia,* and *acinetobacter baumannii* as novel potential biomarkers for oral cancer. These bacteria, particularly *acinetobacter calcoaceticus* and *acinetobacter baumannii*, are known for drug resistance and inflammatory damage, commonly found in healthcare settings. Their increased expression in the mouth could signal a high risk for malignancies, necessitating timely testing or treatment (23, 26, 27). Tumor-associated bacteria present challenges in chemotherapy due to their multi-drug resistance and ability to survive within tumor cells. For instance, *Gammaproteobacteria*, highly expressed in TSCC patients, produce cytidine deaminase (CDD) that inactivates gemcitabine, contributing to chemotherapy resistance (11). These findings highlight the significance of microbiota in cancer treatment and resistance.

An important question raised by our findings is whether the bacterial taxa enriched in TSCC tissues and saliva play a causal role in tumorigenesis or merely represent opportunistic colonizers that thrive within the altered tumor microenvironment. Several of the identified species possess biological properties that could, at least in principle, contribute to cancer development. *Acinetobacter baumannii* and *Acinetobacter calcoaceticus* are known to produce lipopolysaccharide and other pro-inflammatory mediators capable of activating Toll-like receptor signaling, potentially fostering a chronic inflammatory milieu conducive to malignant transformation. *Burkholderia cepacia* can secrete various virulence factors that may interfere with host cell signaling and immune responses. These characteristics suggest that these bacteria might not be passive bystanders but could actively participate in shaping a tumor-promoting microenvironment. Conversely, the tumor microenvironment itself—characterized by hypoxia, immune suppression, and altered nutrient availability—may selectively favor the colonization of specific bacterial species that are otherwise kept in check by intact immune surveillance in healthy tissues. The cross-sectional design of the present study does not allow us to distinguish between these two scenarios. Longitudinal studies tracking microbial changes during the progression from precancerous lesions to invasive carcinoma, as well as experimental models such as gnotobiotic animals or *in vitro* co-culture systems, will be necessary to establish the directionality and causality of the observed microbial associations.

Our study revealed significant differences in the saliva microbiota of TSCC patients compared to normal individuals. TSCC patient saliva showed high abundance of bacteria such as *weissella cibaria*, *sphingomonas azotifigens*, and *aeromonas sobria*, which were rare in normal saliva. Conversely, normal saliva had higher levels of *streptococcus salivarius*, *haemophilus parainfluenzae*, and other species not prevalent in TSCC saliva. These findings suggest that *weissella cibaria*, *sphingomonas azotifigens*, and *aeromonas sobria*. Could serve as potential biomarkers for early, non-invasive oral cancer screening. Liquid biopsy emerges as a promising method due to its convenience, cost-effectiveness, and suitability for early detection, enabling timely intervention and prevention in high-risk individuals.

Several aspects of this study warrant careful consideration. First, the microbial profiles of TSCC patients were compared with healthy reference data obtained from the eHOMD database rather than with a control group prospectively recruited from our own institution. While this approach allowed an initial exploration, it may introduce variability related to population demographics, sampling procedures, and sequencing methodologies. An ideal design would incorporate healthy volunteers or patients with benign oral conditions from the same clinical setting; however, obtaining oral tissue biopsies from healthy individuals presents ethical and practical challenges, and the microbiota associated with benign lesions might not fully represent a normal baseline. These considerations should be kept in mind when interpreting the present findings. Second, the cohort of 24 TSCC patients is modest in size, which naturally limits the statistical power and the breadth of conclusions that can be drawn. The stringent inclusion and exclusion criteria, together with the need for paired tissue and saliva samples and immediate exosome isolation, contributed to a gradual enrollment process. Reassuringly, the main microbial differences reached statistical significance, and several of the highlighted taxa have been noted in earlier studies for their potential relevance to tumor biology. We have therefore framed this work as a pilot investigation, emphasizing hypothesis generation and biomarker discovery rather than clinical validation. Third, although care was taken to exclude major medical confounders—such as other oral diseases, diabetes, hypertension, and recent use of antibiotics or anticancer therapies—certain lifestyle factors known to influence the oral microbiome, including smoking, alcohol consumption, and oral hygiene habits, were not systematically documented or statistically adjusted for in the current analysis. As a result, the degree to which the observed microbial shifts are independent of these behavioral variables remains an open question. Future studies would benefit from larger, prospectively designed cohorts that include in-hospital healthy and benign lesion controls, incorporate standardized collection of lifestyle information, and apply multivariate models to further evaluate the candidate biomarkers identified here.

## Data Availability

The data presented in this study contain potentially identifiable information from human participants and cannot be shared in open-access repositories for legal and ethical reasons. The data are deposited in a restricted-access repository at West China Hospital of Stomatology, Sichuan University, and access can be granted to qualified researchers upon reasonable request to the corresponding author, subject to approval by the institutional ethics committee.

## Footnotes

1
https://www.homd.org/
