# Supplementary material for: Tongue cancer microbial biomarkers: landscape of saliva, cancer tissue, and para-carcinoma tissue
Source: Front Med (Lausanne). 2026 Jun 4;13:1817840. doi: 10.3389/fmed.2026.1817840 (PMC13275251; doi:10.3389/fmed.2026.1817840)

**Supplementary Table 1. baseline characteristics of subjects (N=24)**

| No. | Gender | Age | Diagnosis | TNM staging | Differentiation | Lymphatic metastasis |
| --- | --- | --- | --- | --- | --- | --- |
| 01 | Male | 52 | TSCC | T2N0M0 II | 2 | No |
| 02 | Male | 70 | SCC at the base of the tongue | T3N2bM0 IVA | 2 | Yes |
| 03 | Male | 65 | TSCC | T3N3bM0 IVB | 2 | Yes |
| 04 | Male | 45 | TSCC | T2N0M0 II | 2 | No |
| 05 | Male | 63 | TSCC | T2N0M0 II | 1 | No |
| 06 | Female | 41 | TSCC | T3N0M0 III | 2 | No |
| 07 | Male | 69 | TSCC | cT4aN0M0 IVA | 2 | No |
| 08 | Female | 77 | TSCC | T3N0M0 III | 1 | No |
| 09 | Female | 55 | TSCC | T1N0M0 I | 2 | No |
| 10 | Female | 72 | TSCC | cT3N2cM0 IVA | - | Yes |
| 11 | Male | 54 | SCC of the tongue and floor of the mouth | cT3N2cM0 IVA | 0 | Yes |
| 12 | Female | 72 | TSCC | T2N0M0 II | 3 | No |
| 13 | Male | 68 | TSCC | T2N0M0 II | 2 | No |
| 14 | Female | 32 | TSCC | T2N0M0 II | 2 | No |
| 15 | Male | 48 | TSCC | cT3N0M0 III | 1 | No |
| 16 | Female | 70 | TSCC | T3N0M0 III | 2 | No |
| 17 | Male | 54 | TSCC | T1N0M0 Ⅰ | 1 | No |
| 18 | Female | 69 | TSCC | T1N0M0 Ⅰ | 0 | No |
| 19 | Male | 60 | TSCC | T3N0M0 III | 2 | No |
| 20 | Male | 32 | TSCC | T2N0M0 II | 1 | No |
| 21 | Male | 51 | TSCC | T1N0M0 Ⅰ | 2 | No |
| 22 | Female | 66 | TSCC | cT2N2bM0 IVA | 1 | Yes |
| 23 | Female | 67 | TSCC | T2N0M0 II | 2 | No |
| 24 | Female | 82 | TSCC | T2N0M0 II | 2 | No |

TSCC: Tongue squamous cell carcinoma; SCC: squamous cell carcinoma

Differentiation: original 0, high 1, medium 2, low 3

**Supplementary Figure 1. Exosomes from TSCC Patients Subjected to LEfSe.**

A-B: Taxonomic branching map (Cladogram) illustrating the hierarchical distribution of marker bacteria across the three sample groups: CS, CT, and PT.

C-D: LEfSe analysis (LDA≥4) highlighting the specific distribution of marker bacteria within the CS, CT, and PT groups.


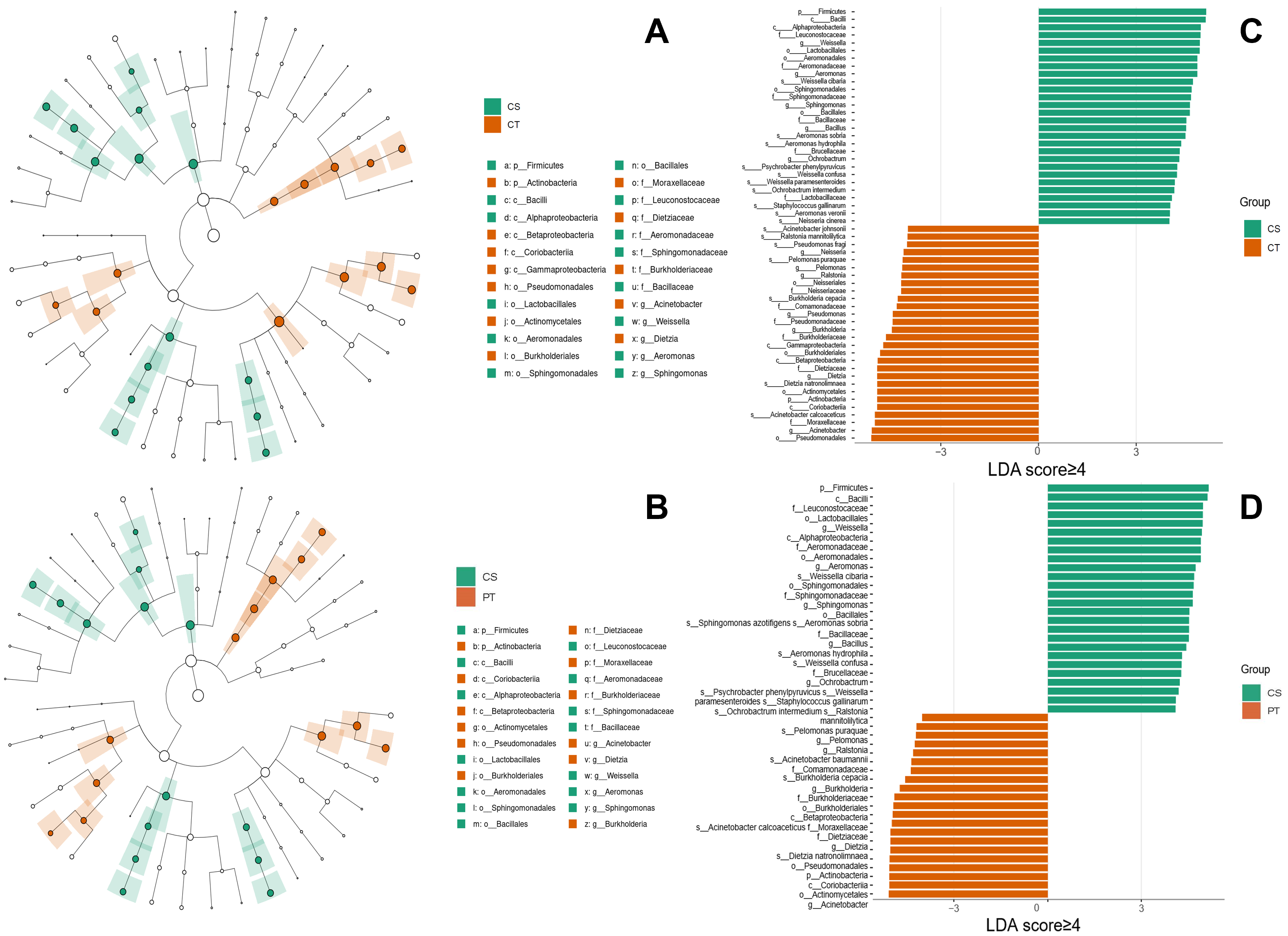


**Supplementary Figure 2. Tax4Fun Functional Prediction of Exosomal Microbiota in TSCC.**


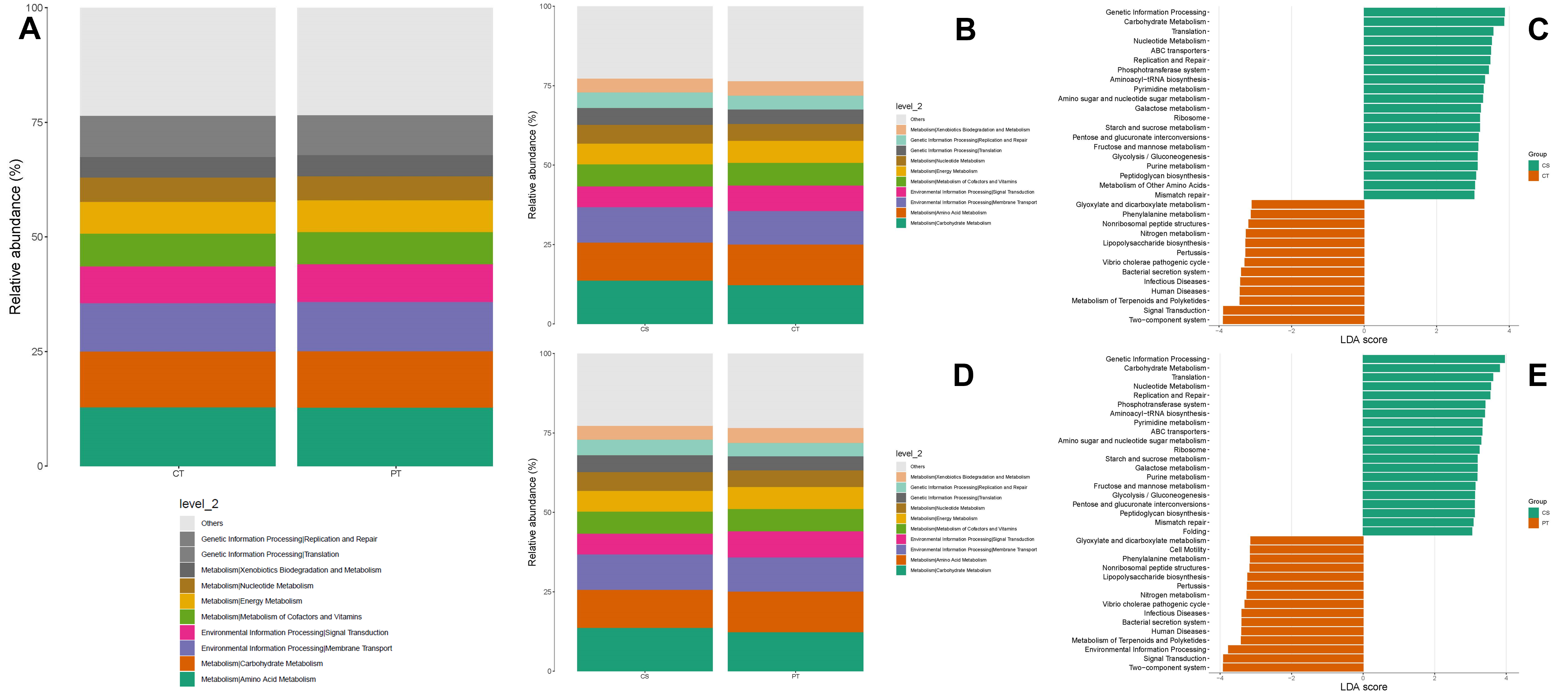

Supplement: Supplementary file 1 [file Data_Sheet_1.docx]
